# Social evaluative implications of sensory adaptation to human voices

**DOI:** 10.1098/rsos.231348

**Published:** 2024-03-27

**Authors:** Kelsey L. Neuenswander, Grace S. R. Gillespie, David J. Lick, Gregory A. Bryant, Kerri L. Johnson

**Affiliations:** ^1^ Department of Communication, University of California, Los Angeles, CA 90095, USA; ^2^ Department of Psychology, University of California, Los Angeles, CA, USA; ^3^ Google, New York City, NY, USA

**Keywords:** sensory adaptation, vocal characteristics, evaluative aftereffects, gender typicality, impression formation

## Abstract

People form social evaluations of others following brief exposure to their voices, and these impressions are calibrated based on recent perceptual experience. Participants adapted to voices with fundamental frequency (*f*_o_; the acoustic correlate of perceptual pitch) manipulated to be gender-typical (i.e. masculine men and feminine women) or gender-atypical (i.e. feminine men and masculine women) before evaluating unaltered test voices within the same sex. Adaptation resulted in contrastive aftereffects. Listening to gender-atypical voices caused female voices to sound more feminine and attractive (Study 1) and male voices to sound more masculine and attractive (Study 2). Studies 3a and 3b tested whether adaptation occurred on a conceptual or perceptual level, respectively. In Study 3a, perceivers adapted to gender-typical or gender-atypical voices for both men and women (i.e. adaptors pitch manipulated in opposite directions for men and women) before evaluating unaltered test voices. Findings showed weak evidence that evaluations differed between conditions. In Study 3b, perceivers adapted to masculinized or feminized voices for both men and women (i.e. adaptors pitch manipulated in the same direction for men and women) before evaluating unaltered test voices. In the feminized condition, participants rated male targets as more masculine and attractive. Conversely, in the masculinized condition, participants rated female targets as more feminine and attractive. Voices appear to be evaluated according to gender norms that are updated based on perceptual experience as well as conceptual knowledge.

## 1. Introduction

People form impressions of others based on minimal information [1]. While most work supporting this conclusion has focused on judgements based on visual information [2], information from other sensory modalities also affects social evaluations [3,4]. For example, perceivers evaluate trustworthiness, dominance and attractiveness from voices alone [5–7], and these judgements impact interpersonal behaviour. People desire to affiliate with others whose voices sound attractive, but not unattractive [8], voters prefer male leaders who sound stereotypically masculine [9,10], and voice characteristics from the first 3 s of defence attorneys’ opening statements predict judicial outcomes in the U.S. Supreme Court [11].

Thus, vocal features clearly influence social judgements. Less clear are the psychological processes that underlie valenced evaluations of particular voice characteristics. We argue that sensory adaptation provides a theoretically viable but heretofore underexplored mechanism. Broadly speaking, adaptation refers to the process by which sensory systems habituate to features perceived in the immediate context, resulting in aftereffects that alter subsequent perceptions [12]. For example, after gazing at a downward-moving object, shifting one’s gaze to a stationary object makes the object appear to drift in the opposite direction of the original motion [13]. Adaptation is ubiquitous in human perception, occurring for visual cues ranging from low-level motion information [13] to higher-level cues for facial identity [14] and to social categories [15]. Adaptation also occurs in other sensory modalities, showing similar effects for low-level auditory dimensions (i.e. loudness and pitch [16,17] and higher-level auditory cues to identity [18,19], affect [20,21], sex [22–25] and age [26].

Perceptual aftereffects share many commonalities across sensory domains. For example, aftereffects are generally contrastive in nature, such that adaptation to one end of a continuum (masculine features) causes neutral test stimuli to be experienced as features at the opposite end of that continuum (feminine features [22]). Such contrastive effects imply that sensory cues are coded in relation to a prototype that is calibrated to recently encountered stimulus features [12]. One influential model of person perception posits that social stimuli are coded in a multidimensional space in which each dimension represents a specific stimulus feature in the population [27]. The centre of the space represents the prototypical person, which takes on average values for all dimensions. Perceptual exposure functionally shifts the location of this prototype towards features that characterize the adapting stimuli. Thereafter, new exemplars are coded in relation to the new norm. Although this multidimensional space initially referred to faces, there is evidence that voices are also represefnted in a multidimensional space in which exemplars are coded in relation to the norm [28–30]. This process explains why adaptation to male-typed voices causes an androgynous test voice to be classified as female. Its features fall far from the newly established prototype and are therefore coded as female [26].

Norm-based coding has theoretical implications not only for perceptual judgements (e.g. categorization and perception) but also for evaluative judgements because perceivers tend to evaluate stimuli based on their prototypicality [31]. Across diverse stimuli [32,33], developmental periods [34] and cultural contexts [35] perceivers generally respond more favourably to prototypical category members than to non-prototypical category members.^1^ In auditory perception, people prefer averageness in stimuli such as music [36] and voices that are averaged through morphing are rated as more attractive than original voices [37]. Consequently, sensory adaptation’s impact on category prototypes is also likely to shift evaluative judgements.

Mounting evidence supports this possibility. In several studies, adaptation to digitally compressed faces caused faces with similar distortions to appear normative and therefore attractive [32]. In other work, adaptation to hyper-masculine female faces enhanced evaluations of women with masculine phenotypes by making them appear more normative [38] and even moderated tendencies to categorize faces as sexual minorities (i.e. gay; [39]). Because adaptation to voices similarly shifts the norm or prototype, one would expect that voices would be evaluated differently after adaptation to masculinized or feminized exemplars. Here, we test whether sensory adaptation to voices influences evaluative judgements for women and men.

In this article, we refer to the perception/manipulation of both sex and gender. There are sexually dimorphic differences in *f*_o_ between males and females which are largely shaped by the development of the vocal folds during puberty [40]. Longer and thicker vocal folds vibrate at a lower rate resulting in lower perceived pitch. When we discuss the categories of men and women, we are referring to these sexually dimorphic differences. However, there is also variability of pitch within males and females which is associated with masculinity and femininity (i.e. lower pitch is associated with masculinity and higher pitch is associated with femininity [41]). Because masculinity and femininity are gendered, we refer to this as gender typicality which is consistent with other social perception research [38].

In four studies, we examined whether auditory adaptation compels evaluative aftereffects of voices. We first tested whether adaptation to gender-typical voices versus gender-atypical voices changed the perceived vocal norms and attractiveness of natural voices of women (Study 1) and men (Study 2). Then, in two studies, we probed whether the effects observed in Studies 1 and 2 were obtained when men’s and women’s voices were combined to shed light on two possible mechanisms. One possibility is that adaptation shifts norms at a conceptual level of gender typicality in which cognitive representations of gender-typical traits (e.g. low-pitched voice for men and high-pitched voice for women) influence evaluations of subsequent voices. Indeed, some evidence suggests that adaptation may operate on a higher conceptual level that transcends sensory modality by showing that adaptation to voice pitch alters subsequent face perception [23]. If this is the case, we would expect similarly favourable evaluations of unaltered voices of men and women after adaptation to typical or atypical adaptors, despite the pitch of these adaptors being directionally opposite. Therefore, in Study 3a, we tested whether adaptation to gender-typical voices versus gender-atypical voices (i.e. directionally opposite pitch manipulations for men and women) might impact normative and evaluative judgements of men’s and women’s natural voices in a similar fashion. If correct, adaptation to gender-typical voices might simultaneously lower evaluative judgements of both men’s and women’s natural voices because they would be perceived as less normative and therefore less attractive. Conversely, adaptation to gender-atypical voices might simultaneously enhance judgements of both men’s and women’s natural voices because they would be perceived as more normative and therefore more attractive.

An alternative possibility is that adaptation shifts norms at a lower *perceptual* level by shifting what is perceived to be the normative pitch more generally. Thus, in Study 3b, we tested the possibility that adaptation to voices operates at a lower perceptual level that is sensitive to the overall direction of the adapting voices. If correct, adaptation to feminized voices of men and women might simultaneously elevate evaluations of men’s but lower evaluations of women’s natural voices, and adaptation to masculinized voices of men and women might simultaneously lower evaluations of men’s but elevate evaluations of women’s natural voices. In Study 3a, we shift adaptors for men and women in opposite directions and expect similar effects on evaluative judgements. In Study 3b, we shift adaptors in the same direction and expect opposite effects on evaluative judgements. Taken together, Studies 3a and 3b offer an initial test of whether adaptation to voices operates through conceptual or perceptual mechanisms, respectively.

The following studies use novel methods to test adaptation and its evaluative aftereffects. Although many researchers create adaptor stimuli with voice morph technology to probe auditory adaptation [25,42], we created adapting stimuli by only manipulating *f*_o_. Voice morphing techniques are often used to alter the perception of sex by manipulating several variables simultaneously, including formant frequencies and other spectral components, allowing for naturalistic sounding stimuli when shifting from male to female voice or vice versa. However, *f*_o_ is a well-established sexually dimorphic aspect of voice [43] and is the strongest cue related to gender perception [42]. Importantly, we did not want our adapting stimuli to be perceived as the opposite sex, but rather as extreme gendered exemplars within the same sex category. Furthermore, popular adaptation paradigms consist of a pre- and post-adaptation phase wherein participants rate targets before and after exposure to adaptors. Adaptation aftereffects change exponentially—they get stronger as a function of adaptation time and weaker as a function of test duration [44]. In the visual and auditory literature, some researchers have adopted a new adaptation paradigm to address these concerns in which aftereffects are tested on each trial rather than after a full adaptation phase [21,44]. We adopted a similar approach in which participants were exposed to an adapting stimulus on each trial and their evaluative aftereffects were measured.

## 2. Study 1

### 2.1. Method

#### 2.1.1. Participants

*A priori* power analyses were conducted to determine the necessary sample size using an application developed by [45]; http://jakewestfall.org/two_factor_power/). Analyses were specific to a standardized NCC between-subjects multilevel design with two random intercepts (targets and participants) and one random slope (condition). The letters N and C indicate whether the random factors in each pair are nested or crossed, respectively [45]. The first letter indicates that participants are nested within condition, the second letter indicates that targets are crossed with condition and the third letter indicates that participants and targets are themselves crossed.

Conducting sample size estimates for multilevel models is particularly complex. Multilevel *a priori* power analyses require knowing numerous parameters such as the number of level-one groups (e.g. how many trials seen per participant), the estimated effect size, variance of random effects, covariance of random effects, regression coefficients and the design effect [46–50]. To assess power, two analyses were run to determine an acceptable range of necessary participants. Based on prior adaptation research [38,51], we estimated a medium to large effect size ranging from *B* = 0.34 to *B* = 0.35. Eighty per cent power was our goal given that it is the standard in social and behavioural sciences [52], and because the substantial increase in required participants when exceeding 80% power is prohibitive. To achieve 80% power with α = 0.05 and an effect size *B* = 0.35, a sample size of at least 116 participants is recommended. To detect an effect size of *B* = 0.34, a sample size of 152 participants is recommended. We were conservative and recruited 181 native English-speaking participants through Prolific (www.prolific.com), who were paid $1.50 for their participation in a 10 min study. Twenty-six participants were excluded from analyses for providing identical ratings of all test voices, yielding a final sample of *N* = 155 (age: *M* = 31.23 years, s.d. = 10.65 years; sex: 46.45% female, 52.26% male, 1.29% other; race: 62.58% White, 17.42% Asian, 8.39% Black, 11.61% biracial/other).

#### 2.1.2. Test stimuli

Test stimuli were audio recordings of 20 different young adult women reciting the sentence: ‘Hi, I’m a student at UCLA’. Samples were recorded digitally (M-Audio Microtrack recorder, 16-bit amplitude resolution, 44.1 kHz sampling rate) using an AKG E535 condenser microphone placed approximately 15 cm from the mouth. Stimuli were normalized in loudness to peak amplitude but varied naturally in duration, fundamental frequency (*f*_o_) and voice quality (table 1).

**Table 1.** Descriptive statistics of the fundamental frequency (Hz) and speech rate (syllables per second) of unaltered women’s voices (test voices).

test voice	*f*_o_	*f*_o_ *s.d.*	*f*_o_ min	*f*_o_ max.	syllables/second
1	185	40	105	272	5.20
2	184	11	156	224	5.84
3	183	20	154	257	5.84
4	195	22	110	244	6.09
5	203	24	100	263	7.10
6	207	11	179	245	4.72
7	189	24	106	254	4.79
8	188	28	139	268	4.56
9	198	41	91	291	4.80
10	200	26	165	255	5.74
11	193	23	151	250	4.61
12	179	23	101	224	6.29
13	197	28	151	260	5.07
14	200	25	149	260	4.11
15	197	31	100	281	5.30
16	214	36	169	294	4.76
17	197	31	148	281	4.57
18	197	19	150	250	4.48
19	181	20	108	252	5.17
20	189	20	104	257	5.52
*M*	194	25	132	259	5.23

#### 2.1.3. Adapting stimuli

Adaptors included both masculinized and feminized exemplars that would be gender-atypical and gender-typical relative to women. Study 1 adaptors were generated from recordings of five young adult women and produced the same sentence as the test stimuli. The decision of which voices were used as test versus adaptor voices was randomized. These recordings were manipulated to be more masculine or feminine using the VT-Change script (C. Darwin) in Praat [53]. We altered *f*_o_ using PSOLA (Pitch Synchronous Overlap Add) resynthesis. The average female voice is approximately 200 Hz [40], although there is substantial variability and range. For masculinized (i.e. gender-atypical) versions, *f*_o_ values were systematically lowered to reach the ‘atypical’ range (i.e. still perceived as female but lower pitched than the average). Thus, voices were manipulated down in pitch by either 65% or 75% of baseline to accommodate natural variation in the unaltered recordings. For feminized (i.e. gender-typical) versions, *f*_o_ was increased to 130% of baseline, and all adaptors could be manipulated up in pitch the same amount to be considered in the ‘typical’ range.^2^ Thus, although there was still natural variability in atypical and typical adaptors, the voices within each adaptor category were all similar in pitch (table 2).

**Table 2.** Descriptive statistics of the fundamental frequency (Hz) and speech rate (syllables per second) of women’s voices (adaptors) pitch-adjusted up (top panel) and pitched-adjusted down (bottom panel).

adaptor	*f*_o_	*f*_o_ s.d.	*f*_o_ min	*f*_o_ max.	syllables/second
1	254	31	128	301	5.61
2	239	28	116	300	6.31
3	224	25	171	299	4.86
4	260	40	107	300	4.85
5	254	27	167	295	5.83
*M*	246	30	138	299	5.49
1	138	12	109	167	5.61
2	129	17	100	193	6.31
3	129	14	103	173	4.86
4	144	15	117	174	4.85
5	132	13	103	172	5.83
*M*	134	14	106	176	5.49

#### 2.1.4. Procedure

Prior to the study, participants confirmed that they were wearing headphones and in a quiet environment. Participants were then randomly assigned to either a gender-typical (feminized) or gender-atypical (masculinized) adaptation condition for women’s voices^3^. On each trial, participants first heard an adapting voice followed by a test voice which they judged for attractiveness and femininity (1 = *not at all* to 9 = *extremely*). Judgements of likeability, typicality and friendliness were also collected (1 = *not at all* to 9 = *extremely*). These exploratory judgements were collected to inform future research and thus are not reported in the current manuscript. To maintain attention, participants completed a secondary task rating whether the pitch of each adaptor was higher, lower or identical to the previous voice. In total, participants completed 20 trials in pseudo-randomized order, with each adaptor presented four times. After participants completed these trials, they verified again that they wore headphones for the duration of the study and reported whether they experienced any audio difficulties.

#### 2.1.5. Transparency and openness

The following studies were approved by the UCLA Institutional Review Board (#20-001311). All participants provided informed consent prior to their participation and were fully debriefed after completion. We report how we determined our sample size, all data exclusions and all manipulations. All data, analysis code for their participation and research materials are available at OSF (osf.io/msnke). Data were collected in 2021 and analysed using R, v.4.1.2 [54]. The following R packages were used: *lme4* (v.1.1-29; [55], *lmerTest* (v.3.1.3 [56]) and *mediation* (v.4.5.0 [57]).

### 2.2. Results

Perceivers evaluate men and women regarding their sexually dimorphic vocal properties, often rating men as more attractive when their voices have low *f*_o_ but women as more attractive when their voices have high *f*_o_ [58,59]. Therefore, we predicted that adaptation to gender-atypical (i.e. masculinized) voices would produce contrastive aftereffects, making neutral test voices sound more gender-typical and therefore more attractive. Conversely, adaptation to gender-typical (i.e. feminized) voices should make neutral test voices sound less typical and therefore less attractive.

To conduct our analyses, we created multilevel random coefficient models with two random intercepts (targets and participants) and one random slope (condition) using the *lme4* (v.1.1-29; [55]) and *lmerTest* (v.3.1.3 [56]); packages in R [54]. Participant sex was initially included in all analyses, but its effect did not moderate the results. As such, it was dropped from these analyses. Furthermore, voice *f*_o_ was initially included as a moderator, but no effects were significant. This was likely due to similar *f*_o_ values across test voices.

The secondary pitch judgement task was used as a manipulation check to determine if participants accurately perceived adaptors as being higher or lower pitched than test voices. Responses to the question ‘Is the pitch of this voice higher, lower or identical to the previous voice?’ were recoded to 1, −1 and 0, respectively. Values above 0 indicate that the adaptor was rated as higher pitched than the test voice and values below 0 indicate that the adaptor was rated as lower pitched than the test voice. We regressed pitch judgement onto adaptation condition. As expected, participants rated atypical adaptors (*M* = −0.43, s.d. = 0.80) as significantly lower pitched than typical adaptors (*M* = 0.49, s.d. = 0.77), *B* = 0.93, s.e. = 0.07 and *t* = 13.68, *p* < 0.001.

**Figure 1.**
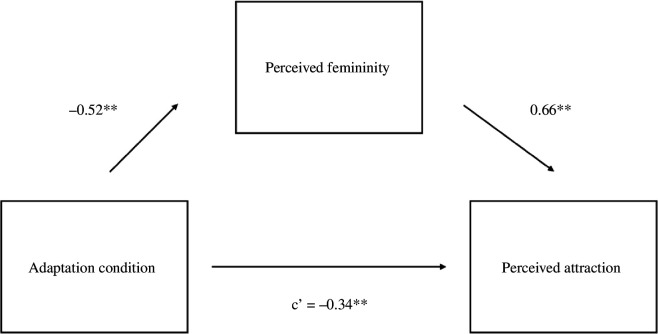
Study 1 Mediation model for women’s voices*.* Note. *p < 0.05, **p < 0.01, ***p < 0.001.

We then tested whether adaptation altered evaluations of female voices by regressing attractiveness onto adaptation condition. Test voices were rated as more attractive after adaptation to gender-atypical voices (*M* = 6.01, s.d. = 1.96) relative to gender-typical voices (*M* = 5.64, s.d. = 1.92), *B* = −0.39, s.e. = 0.18, *t* = −2.15 and *p* = 0.033. Next, we tested whether adaptation altered perceptual norms by regressing perceived femininity onto adaptation condition. Test voices sounded more feminine after adaptation to gender-atypical voices (*M* = 6.30, s.d. = 1.91) relative to gender-typical voices (*M* = 5.80, s.d. = 1.94), *B* = −0.52, s.e. = 0.16 and *t* = −3.23, *p* = 0.002. Finally, we tested whether the shift in perceptual norm accounted for the shift in evaluation using the multilevel *mediation* package in R (v.4.5.0 [57,60]. Results indicated a significant indirect effect of perceived femininity on the association between adaptation condition and attractiveness, *p* = 0.002, 95% CI: −0.55 to −0.12 (figure 1).

## 3. Study 2

### 3.1. Method

#### 3.1.1. Participants

The design and effects of interest for Studies 1 and 2 are identical, so we aimed to recruit a similar number of participants. We successfully exceeded Study 1 sample size with 179 Prolific participants (www.prolific.com) who were paid $1.50 for their participation in a 10 min study. Nine of these participants were excluded from analyses because they provided identical responses to every question. This yielded a final sample of 170 native English-speaking participants (age: *M* = 25.39 years, s.d. = 8.56 years; sex: 80% female, 17.65% male, 2.35% other; race: 67.65% White, 5.88% Asian, 8.24% Black, 18.24% biracial/other).

#### 3.1.2. Test stimuli

Test stimuli were audio recordings of 20 young adult men reciting the same sentence as Study 1 stimuli (table 3).

**Table 3.** Descriptive statistics of the fundamental frequency (Hz) and speech rate (syllables per second) of unaltered men’s voices (test voices).

test voice	*f*_o_	*f*_o_ s.d.	*f*_o_ min	*f*_o_ max.	syllables/seconds
1	112	9	100	145	4.89
2	115	12	98	145	4.67
3	135	7	124	166	5.63
4	109	8	85	131	5.10
5	143	10	122	161	5.28
6	99	8	76	113	5.49
7	110	9	88	138	6.15
8	98	8	80	112	5.05
9	99	10	78	120	5.17
10	96	5	78	113	5.21
11	107	16	78	173	4.31
12	124	21	81	174	5.78
13	113	7	96	126	4.85
14	124	16	84	196	6.27
15	115	10	98	135	5.69
16	126	10	107	145	5.90
17	138	24	80	188	5.18
18	113	7	101	142	4.80
19	102	13	79	134	6.98
20	110	4	98	119	5.95
*M*	114	11	92	144	5.42

#### 3.1.3. Adapting stimuli

Adaptors were generated from recordings of five young adult men producing the same sentence as test stimuli (table 4). The average male voice is approximately 100 Hz [40]. However, like female voices, there is a wide range of vocal pitch for men. Due to natural variation in the unaltered adaptors, some voices had to be pitch-adjusted up more to fall within the ‘atypical’ range. However, all adaptors could be pitch-adjusted down the same amount to be considered in the ‘typical’ range. For masculinized (gender-typical) versions, *f*_o_ values were lowered to 90% of baseline. For feminized (gender-atypical) versions, *f*_o_ was increased to 175% of baseline.

**Table 4.** Descriptive statistics of the fundamental frequency (Hz) and speech rate (syllables per second) of men’s voices (adaptors) pitched-adjusted up (top panel) and pitched-adjusted down (bottom panel).

adaptor	*f*_o_	*f*_o_ s.d.	*f*_o_ min	*f*_o_ max.	syllables/second
1	155	10	135	181	5.44
2	159	12	136	199	6.30
3	164	17	133	193	5.18
4	163	18	132	199	5.49
5	167	11	135	191	7.49
*M*	162	14	134	192	5.98
1	82	5	75	104	5.44
2	83	6	75	104	6.30
3	87	7	75	99	5.18
4	86	8	74	101	5.49
5	87	5	77	100	7.49
*M*	85	6	75	102	5.98

#### 3.1.4. Procedure

Study 2 followed the same paradigm as Study 1 but we used men’s voices rather than women’s voices. Participants were assigned to either gender-typical (i.e. masculinized) or gender-atypical (i.e. feminized) adaptation condition for men and evaluated test voices for attractiveness and masculinity (1 = *not at all* to 9 = *extremely*).

### 3.2. Results

As in Study 1, we predicted that adaptation to gender-atypical (i.e. feminized) voices would produce contrastive aftereffects, making neutral test voices sound more gender-typical and therefore more attractive. Conversely, we expected that adaptation to gender-typical (i.e. masculinized) voices would make neutral test voices sound less typical and therefore less attractive. The main distinction is that the relative pitch considered typical or atypical for men is directionally opposite of what is considered typical or atypical for women.

We regressed pitch judgement onto adaptation condition to check if our adaptation manipulations were successful. As expected, participants rated atypical adaptors (*M* = 0.47, s.d. = 0.79) as significantly higher pitched than typical adaptors (*M* = −0.50, s.d. = 0.74), *B* = −0.97, s.e. = 0.07, *t* = −14.06, *p* < 0.001.

**Figure 2.**
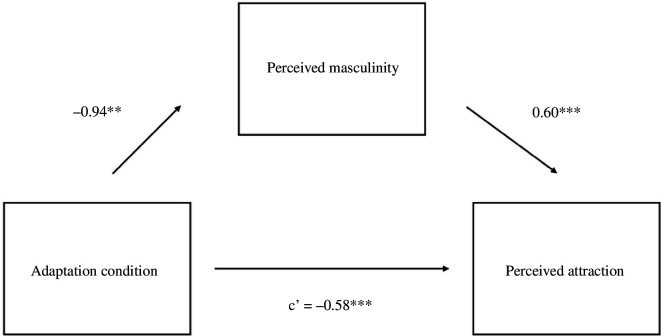
Study 2 Mediation model for men’s voices. Note. *p < 0.05, **p < 0.01, ***p < 0.001.

Furthermore, our results confirmed our predictions. Male test voices in Study 2 were rated as more attractive after adaptation to gender-atypical (i.e. feminized) voices (*M* = 5.57, s.d. = 2.16) relative to gender-typical (i.e. masculinized) voices (*M* = 4.96, s.d. = 2.14), *B* = −0.61, s.e. = 0.17, *t* = −3.54, *p* <0.001. Test voices were also judged as being more masculine after adaptation to gender-atypical voices (*M* = 5.92, s.d.= 1.90) relative to gender-typical voices (*M* = 4.98, s.d. = 1.91), *B* = −0.94, s.e. = 0.16, *t* = −5.74 and *p* <0.001. Results indicated a significant indirect effect of perceived masculinity on the association between adaptation condition and attractiveness, *p* < 0.001, 95% CI: −0.77 to −0.38 (figure 2).

### 3.3. Summary

Results from Studies 1 and 2 supported our hypothesis that adaptation to gender-atypical voices causes contrastive aftereffects, and that these aftereffects impact evaluations of typicality and thus attractiveness for both women and men. However, these results do not reveal whether participants adapted to conceptual cues of gender typicality [23] or *perceptual* cues of voice pitch. Although we found comparable effects of gender typicality on aftereffects in Studies 1 and 2, the adaptors considered atypical or typical for women and men were pitch manipulated in opposite directions. For women, atypical and typical voices were pitch-adjusted down and up, respectively. For men, the opposite was true. It is possible that evaluations of voices were impacted by listeners’ knowledge of gender typicality rather than solely by the perceptual features of the voice. Although we expect aftereffects to arise quite quickly (i.e. on each trial), we also expect aftereffects to strengthen as a function of adaptation time throughout the experiment [44]. Therefore, if participants demonstrate strong aftereffects after they are exposed to directionally opposed adaptors throughout the experiment, this would suggest that the concept of gender typicality is driving judgements. In contrast, if aftereffects are diminished when listeners adapt to directionally opposed adaptors, this would suggest that pitch is driving judgements.

Studies 3a and 3b offer initial tests between these possibilities by measuring whether the observed effects adjust perceived norms and evaluative judgements at a higher conceptual level by shifting norms for perceived gender typicality (Study 3a) or at a lower perceptual level by shifting norms for perceived average pitch (Study 3b). Importantly, these studies each provide a unique contribution for distinguishing between two possible adaptation patterns, one that simultaneously shifts gendered expectations, broadly (Study 3a), and another that shifts more local perceptual norms, specifically (Study 3b).

## 4. Study 3a

### 4.1. Method

#### 4.1.1. Participants

We ran an additional power analysis to account for added targets that were judged by participants (40 versus 20). To achieve 80% power with a relatively large effect size of *B* = 0.35, a minimum of 59 participants is recommended. Although our effect sizes were large in Studies 1 and 2, we ran an additional power analysis with a smaller critical effect size of *B* = 0.30 in the case that the longer study design muted any of our results. This analysis recommended 101 participants. Given that our previous studies required several participants to be excluded from analyses, we were conservative and recruited 178 native English-speaking participants from Prolific (www.prolific.com) who were paid $3.00 for their participation in a 20 min study. Fifteen participants were excluded for providing identical responses across all trials, leaving a final sample size of *N* = 168 (age: *M* = 25.60 years, s.d. = 7.62 years; sex: 75% female, 22.02% male, 2.98% other; race: 63.10% White, 10.71% Asian, 9.52% Black, 16.67% biracial/other).

#### 4.1.2. Test stimuli

Study 3a used all test stimuli from Studies 1 and 2, yielding a total of 40 test voices (20 women and 20 men) in each study.

#### 4.1.3. Adapting stimuli

Adaptors from both Studies 1 (five women) and 2 (five men) were used.

#### 4.1.4. Procedure

Participants confirmed that they were wearing headphones and in a quiet environment before they were randomly assigned to either a gender-typical or gender-atypical condition. Each participant heard a block of female voices and a block of male voices in counterbalanced order. Thus, participants in the gender-typical condition heard feminized adaptors before making judgements about female test voices and heard masculinized adaptors before making judgements about male test voices. Conversely, participants in the gender-atypical condition heard masculinized adapters before making judgements about female test voices and heard feminized adaptors before making judgements about male test voices. On each trial, participants first heard an adapting voice, followed by a test voice which they judged for attractiveness and masculinity/femininity. Participants also completed a secondary task in which they rated whether the adapting voice was higher, lower or the same pitch as the previous voice. After participants completed these trials, they verified again that they wore headphones for the duration of the study and reported whether they experienced any audio difficulties.

### 4.2. Results

We regressed pitch judgements onto adaptation condition and target sex. There was a significant interaction, *B* = 1.78, s.e. = 0.07, *t* = 25.88 and *p* < 0.001. Tests of simple effects revealed that when centred on male voices, typical adaptors (*M* = −0.46, s.d. = 0.76) were rated as significantly lower pitched than atypical adaptors (*M* = 0.39, s.d. = 0.81), *B* = 0.85, s.e. = 0.05, *t* = 15.51, *p* < 0.001. When centred on female voices, typical adaptors (*M* = 0.53, s.d. = 0.72) were rated as significantly higher pitched than atypical adaptors (*M* = −0.41, s.d. = 0.81), *B* = −0.94, s.e. = 0.05, *t* = −17.10, *p* < 0.001. This suggests that our adaptors were successfully manipulated.

**Figure 3.**
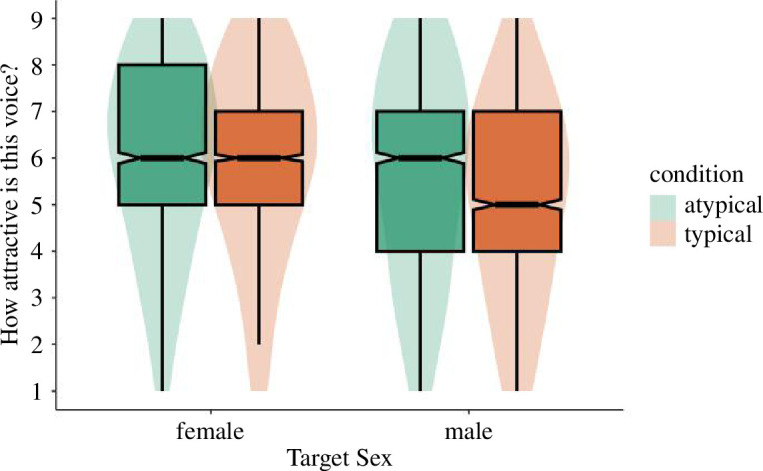
Study 3a perceived attractiveness by adaptation condition and target sex.

If participants adapt to conceptual cues of gender typicality, then we would expect there to be a main effect of adaptation condition on attractiveness (regardless of target sex). We first tested whether adaptation altered evaluations of test voices by regressing attractiveness onto adaptation condition and target sex. Although trending in the predicted direction, the effect of adaptation condition on perceived attractiveness did not reach significance, *B* = −0.29, s.e. = 0.17, *t* = −1.69, *p* = 0.093 (atypical: *M* = 5.84, s.d. = 2.16; typical: *M* = 5.55, s.d. = 2.12). There was a main effect of target sex on attractiveness, with female test voices (*M* = 5.96, s.d. = 2.05) rated as significantly more attractive than male test voices (*M* = 5.41, s.d. = 2.20), *B* = −0.67, s.e. = 0.21, *t* = −3.23, *p* = 0.003. Furthermore, there was a significant interaction between adaptation condition and target sex, *B* = −0.44, s.e. = 0.08, *t* = −5.19, *p* < 0.001 (figure 3). Simple effects revealed that the effect of adaptation condition did not significantly impact judgements of female targets (atypical: *M* = 6.00, s.d. = 2.07; typical: *M* = 5.93, s.d. = 2.04), *B* = −0.07, s.e. = 0.17, *t* = −0.39, *p* = 0.695. However, male targets were rated as more attractive after adaptation to atypical (*M* = 5.68, s.d. = 2.24) rather than typical (*M* = 5.17, s.d. = 2.13) voices, *B* = −0.51, s.e. = 0.17, *t* = −2.91, *p* = 0.004. This provides some support for conceptual adaptation that is stronger for men’s voices than women’s.

**Figure 4.**
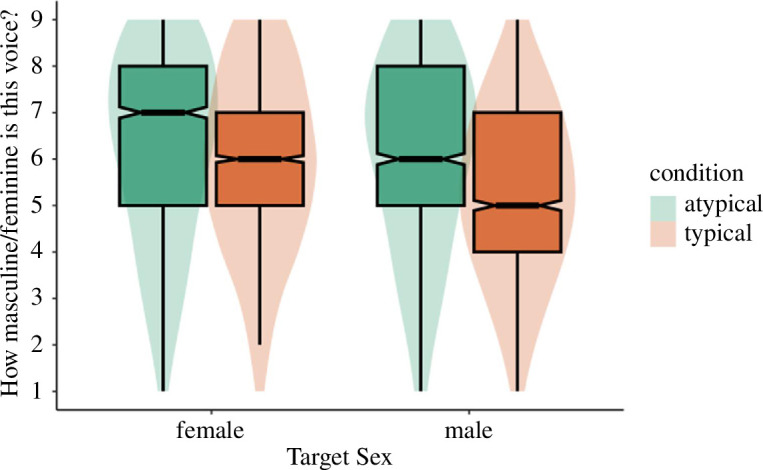
Study 3a perceived masculinity/femininity by adaptation condition and target sex.

**Figure 5.**
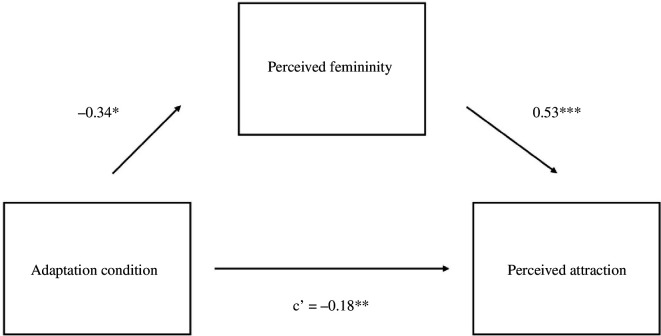
Study 3a Mediation model for female targets. Note. *p < 0.05, **p < 0.01, ***p < 0.001.

**Figure 6.**
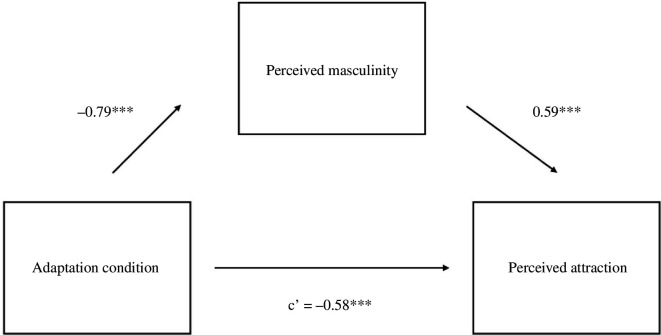
*S*tudy 3a Mediation model for male targets. Note. *p < 0.05, **p < 0.01, ***p < 0.001.

Next, we tested whether adaptation altered perceptual norms by regressing perceived femininity/masculinity onto adaptation condition and target sex. If participants are sensitive to conceptual cues of gender typicality we would expect a main effect of adaptation condition on femininity/masculinity ratings despite the pitch of typical male and female adaptors being directionally opposed. The results supported this. Test voices were rated as more feminine/masculine after adaptation to gender-atypical voices (*M* = 6.15, s.d. = 2.05) relative to gender-typical voices (*M* = 5.59, s.d. = 1.91), *B* = −0.57, s.e. = 0.15, *t* = −3.67, *p* < 0.001. There was a significant effect of target sex on perceived femininity/masculinity, such that female test voices (*M* = 6.06, s.d. = 1.99) were rated as significantly more feminine than male test voices were rated masculine (*M* = 5.64, s.d. = 1.98), *B* = −0.85, s.e. = 0.23, *t* = −3.67, *p* < 0.001. Furthermore, the interaction between condition and target sex was significant, *B* = −0.45, s.e. = 0.08, *t* = −5.60, *p* < 0.001 (figure 4). For judgements of female targets, female test voices were rated as more feminine after adaptation to gender-atypical (*M* = 6.24, s.d. = 2.05) relative to gender-typical voices (*M* = 5.90, s.d. = 1.92), *B* = −0.34, s.e. = 0.16, *t* = −2.20, *p* = 0.029. A similar but stronger effect was found for judgements of male targets. Male test voices were rated as more masculine after adaptation to gender-atypical (*M* = 6.06, s.d. = 2.05) relative to gender-typical voices (*M* = 5.27, s.d. = 1.84), *B* = −0.79, s.e. = 0.16, *t* = −5.06, *p* < 0.001. We ran mediation models separately for male and female targets. In both cases, the effect of adaptation condition on attraction ratings was fully mediated via perceived femininity and masculinity, respectively (figures 5 and 6). The indirect effect for female targets was −0.18: *p* = 0.048, 95% CI: −0.34 to −0.00. The indirect effect for male targets was −0.48: *p* < 0.001, 95% CI: −0.66 to −0.29.

Taken together, the results for Study 3a provided some support for the possibility that adaptation shifts norms at a conceptual level. Judgements of attractiveness, although directionally consistent with the possibility that adaptation shifts norms at a conceptual level, did not reach significance for female targets. However, adaptation significantly shifted perceptions of masculinity/femininity which impacted attractiveness judgements. We next test the possibility that adaptation might impact evaluative judgements by shifting perceptual norms of average pitch, rather than shifting conceptual norms of gender typicality.

## 5. Study 3b

### 5.1. Method

#### 5.1.1. Participants

The same justification for Study 3a sample size was used to recruit participants in Study 3b. A total of 178 participants were recruited from Prolific ( www.prolific.com) who were compensated $3.00 for their participation in a 20 min study. Three participants were excluded for providing identical responses to all questions. This led to a final sample of 175 native English-speaking individuals (age: *M* = 25.36 years, s.d. = 7.01 years; sex: 76.57% female, 21.71% male, 1.71% other; race: 60.57% White, 8.57% Asian, 9.71% Black, 21.24% biracial/other).

#### 5.1.2. Stimuli and procedure

Study 3b stimuli were identical to Study 3a. However, rather than being assigned to a typical or atypical adaptation condition, participants were randomly assigned to either a masculinized or feminized condition. Similar to Study 3a, each participant heard a block of female voices and a block of male voices. Thus, participants in the masculinized condition heard masculinized adaptors before making judgements about female voices and heard masculinized adaptors before making judgements about male test voices. Participants in the feminized condition heard feminized adaptors before making judgements about females and heard feminized adaptors before making judgements about male test voices. The same dependent measures as Study 3a were collected.

### 5.2. Results

We regressed pitch judgements onto adaptation condition and target sex. Because the direction of adaptors was consistent for both male and female voices, we did not expect a significant interaction. Our results confirmed this, *B* = 0.04, s.e. = 0.08, *t* = 0.54, *p* = 0.594. Indeed, there was a significant main effect of condition, *B* = −0.99, s.e. = 0.07, *t* = −14.58, *p* < 0.001. Participants in the feminized condition (*M* = 0.48, s.d. = 0.76) rated adaptors as significantly higher pitched than participants in the masculinized condition (*M* = −0.48, s.d. = 0.75).

Because participants were exposed to both gender-typical and gender-atypical adaptors in Study 3b, we no longer expected a main effect of adaptation condition. Rather, if participants adapted to perceptual cues of voice pitch, we expected the interaction between adaptation condition and target sex to be significant. In other words, participants in the masculinized condition were expected to rate male test voices as less attractive than participants in the feminized condition, and female test voices as more attractive than participants in the feminized condition. Conversely, participants in the feminized condition were expected to rate male test voices as more attractive than participants in the masculinized condition, and female test voices as less attractive than participants in the masculinized condition.

**Figure 7.**
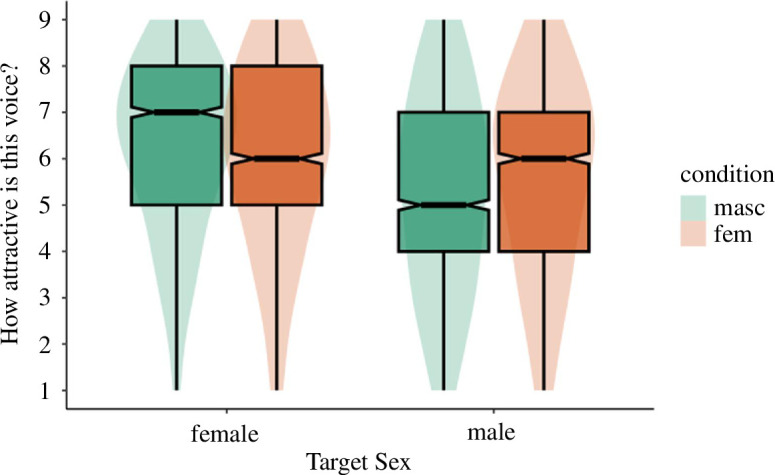
Study 3b perceived attractiveness by adaptation condition and target sex.

We tested whether adaptation altered evaluations of test voices by regressing attractiveness onto adaptation condition and target sex. The effect of adaptation condition on perceived attractiveness was not significant, *B* = 0.06, s.e. = 0.16, *t* = 0.40, *p* = 0.693. Participants rated test voices as similarly attractive after adaptation to masculinized voices (*M* = 5.76, s.d. = 2.09) and feminized voices (*M* = 5.82, s.d. = 2.06). Like Study 3a, female test voices (*M* = 6.13, s.d. = 1.94) were rated as significantly more attractive than male test voices (*M* = 5.45, s.d. = 2.15), *B* = −0.85, s.e. = 0.21, *t* = −4.10, *p* < 0.001. Importantly, the interaction between adaptation condition and target sex was significant, *B* = 0.58, s.e. = 0.08, *t* = 7.14, *p* < 0.001 (figure 7). Tests of simple effects showed that for female targets, there was no significant difference in attractiveness ratings after adaptation to feminized (*M* = 6.02, s.d. = 2.00) or masculinized adaptors (*M* = 6.24, s.d.= 1.87), *B* = −0.23, s.e. = 0.16, *t* = −1.45, *p* = 0.148. However, for judgements of male targets, male test voices were rated as significantly more attractive after adaptation to feminized (*M* = 5.63, s.d. = 2.10) relative to masculinized adaptors (*M* = 5.28, s.d. = 2.18), *B* = 0.35, s.e. = 0.16, *t* = 2.25, *p* = 0.026. This replicates the pattern of results found in Study 3a.

**Figure 8.**
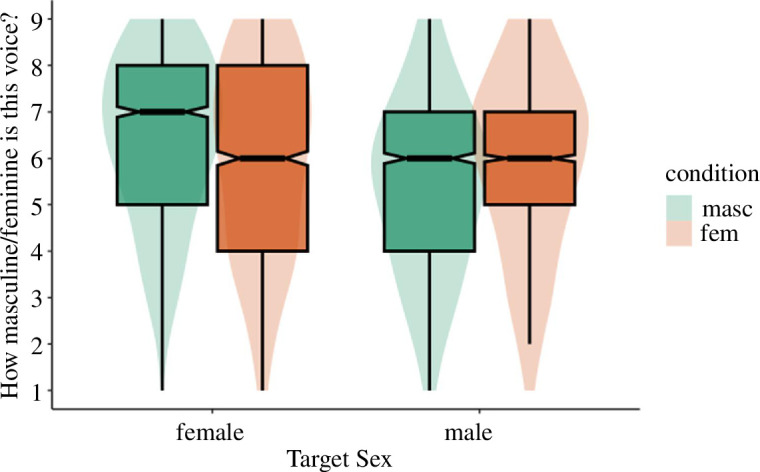
Study 3b perceived masculinity/femininity by adaptation condition and target sex.

**Figure 9.**
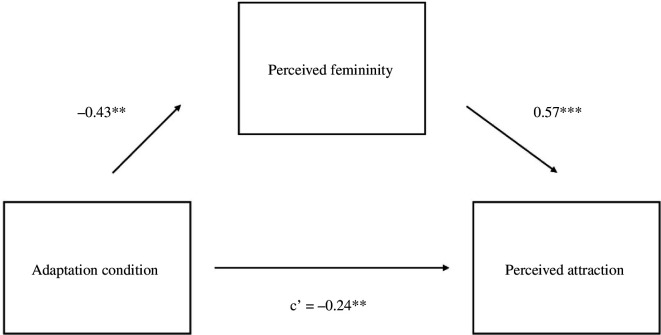
Study 3b Mediation model for female targets. Note. *p < 0.05, **p < 0.01, ***p < 0.001.

**Figure 10.**
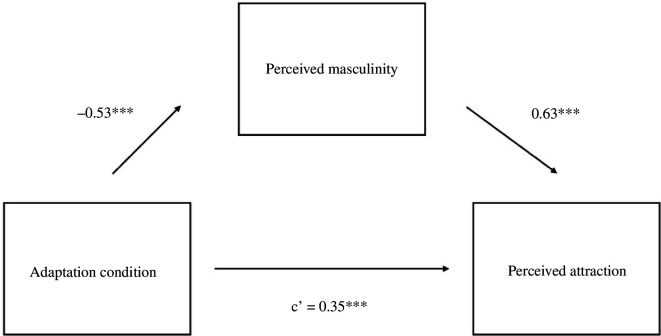
Study 3b Mediation model for male targets. Note. *p < 0.05, **p < 0.01, ***p < 0.001.

Next, we regressed perceived femininity/masculinity onto adaptation condition and target sex. There was no effect of adaptation condition on ratings of femininity/masculinity (feminized: *M* = 5.95, s.d. = 2.00; masculinized: *M* = 5.91, s.d. = 1.89), *B* = 0.05, s.e. = 0.16, *t* = 0.30, *p* = 0.768. Again, there was an effect of target sex such that female voices (*M* = 6.22, s.d. = 1.93) were rated as significantly more feminine than male voices (*M* = 5.64, s.d. = 1.91) were rated masculine, *B* = −1.30, s.e. = 0.23, *t* = −5.62, *p* < 0.001. Finally, there was a significant interaction between adaptation condition and target sex, *B* = 0.96, s.e. = 0.16, *t* = 5.91, *p* < 0.001 (figure 8). Tests of simple effects revealed that male test voices were rated as significantly more masculine after adaptation to feminized voices (*M* = 5.90, s.d. = 1.94) versus masculinized voices (*M* = 5.37, s.d. = 1.85), *B* = 0.52, s.e. = 0.16, *t* = 3.31, *p* = 0.001. Conversely, female test voices were rated as significantly more feminine after adaptation to masculinized voices (*M* = 6.44, s.d. = 1.78) versus feminized voices (*M* = 6.00, s.d. = 2.06), *B* = −0.43, s.e. = 0.16, *t* = −2.73, *p* = 0.007. For both female and male targets, the effect of adaptation condition on attraction ratings was fully mediated via perceived femininity and masculinity, respectively (figures 9 and 10). The indirect effect for female targets was −0.24: *p* < 0.001, 95% CI: −0.40 to −0.08. The indirect effect for male targets was 0.35: *p* = 0.002, 95% CI: 0.13 to 0.56.

In sum, there is evidence to suggest that the process of auditory adaptation happens on a perceptual level, as most existing research attests, which has downstream effects on perceptions of typicality and thus attractiveness.

## 6. General discussion

Four studies revealed that evaluative preferences for voices were calibrated based on recent sensory exposure. Perceivers adapted to women’s voices (Study 1), men’s voices (Study 2) or both (Studies 3a and 3b) that were systematically manipulated to be gender-typical or gender-atypical. A summary of our manipulations and findings is presented in table 5. Relative to gender-typical cues, adaptation to gender-atypical cues caused test voices to sound more attractive by making them sound more typical (Studies 1 and 2). There is some evidence that evaluative aftereffects were a result of both conceptual adaptation to gender typicality (Study 3a) and perceptual adaptation to voice pitch (Study 3b); however, the current results suggest that voice pitch appears to have a stronger effect on adaptation and evaluative judgements. Furthermore, aftereffects were stronger for men’s voices than women’s voices.

**Table 5.** Summary of independent variables (IV), dependent variables (DV) and findings for Studies 1–3.

study	voices	IV	DV	findings
1	women only	atypical condition: voices pitch-adjusted down.typical condition: voices pitch-adjusted up.	femininity attractiveness	participants in atypical adaptation condition rated test voices as more feminine and attractive than those in the typical condition.
2	men only	atypical condition: voices pitch-adjusted up.typical condition: voices pitch-adjusted down.	masculinity attractiveness	participants in atypical adaptation condition rated test voices as more masculine and attractive than those in the typical condition.
3a	women and men	atypical condition: Women’s voices pitch-adjusted down and men’s voices pitch-adjusted up.typical condition: Women’s voices pitch-adjusted up and men’s voices pitch-adjusted down.	femininity or masculinity attractiveness	participants in the atypical adaptation condition rated men’s and women’s test voices as more masculine/feminine and slightly more attractive than participants in the typical condition.
3b	women and men	masculinized condition: Women’s and men’s voices both pitch-adjusted down.feminized condition: Women’s and men’s voices both pitch-adjusted up.	femininity or masculinity attractiveness	participants in the masculinized adaptation condition rated men’s test voices as less masculine and attractive, and women’s test voices as more feminine and attractive, than participants in the feminized condition.

These findings support the idea that sensory adaptation is a general mechanism guiding impression formation. Preferences for facial cues are calibrated by visual experience, resulting in favourable evaluations for facial features that resembled those that were encountered recently [38,61]. The current studies expand these observations to include auditory cues, suggesting that perceptual adaptations can and do occur across sensory modalities. Existing research has supported a multidimensional voice space in which exemplars are coded based on recent experience [26,28–30]. We complement and extend these findings by suggesting that there are consequences of this adaptation. Exposure to atypical voices shifted the norm in which subsequent exemplars were evaluated.

Perceived voice pitch and accompanying social evaluations of gender typicality and attractiveness are likely to have numerous real-world implications. Extant evidence suggests that voice pitch influences important outcomes such as electability [9,62–64] and labour market success of male CEOs [65] even though pitch is not a true signal of leadership ability [66]. Male advocates are more likely to win a U. S. Supreme Court case if their voices are perceived as less masculine [11]. Gender-typical voices are perceived as more attractive [41,67] but more likely to commit infidelity than gender-atypical voices [68,69]. Attractive voices are evaluated more favourably on irrelevant traits such as likeability [8,70]. Our findings demonstrate that judgements of voice pitch, gender typicality and attractiveness are not static. Rather, they are influenced by recent perceptual experience. Thus, these important outcomes might also be affected by proximal perceptual exposure.

Recent work failed to find effects consistent with the current studies. Ostrega, Little and Feinberg [71] used a different adaptation paradigm and reported no evidence of adaptation effects on attractiveness judgements. The distinctions between the adaptation paradigms and vocal stimuli used in each set of studies suggest that further investigations are warranted. Based on the current evidence, we maintain that vocal adaptation effects can and do occur, a possibility that is buttressed by parallel findings in adaptation to faces, which share the same underlying coding strategy as voices [29].

### 6.1. Limitations and future directions

These studies provide a foundation for informing future research. First, our manipulation of adaptors was intended to shift gender typicality rather than perceived sex, it remains possible that some stimuli might have been implicitly miscategorized by participants. There is some evidence that adaptation across sex boundaries is difficult to achieve [38,51]. If our adaptors elicited cross-sex categorizations, our consistent pattern of results across studies would have been unlikely to be observed. Additionally, we intentionally focused our participants’ attention on pitch during their incidental encoding task to provide them with a task. Doing so might have unintentionally altered their judgements of attractiveness and typicality, although it remains unlikely that this possibility would lead to systematic patterns across studies. An intriguing possibility for future research is to measure the perceived pitch of test voices, rather than the adaptors, to more explicitly test if the perceived pitch of unmanipulated voices shifts as a function of exposure to adaptors. Measuring the perceived pitch of test voices would also eliminate the possibility that our findings are due to experience-driven calibration of social judgements rather than true perceptual adaptation.

Our findings provide important evidence regarding the relative strength of conceptual versus perceptual mechanisms underlying adaptation. While Studies 3a and 3b replicated our previous findings and began to adjudicate between these possibilities, additional research is warranted. First, conceptual adaptation is inherently linked with perceptual adaptation in our studies given that we manipulated pitch in all cases, a challenge that is common to related research that has attempted to disentangle these mechanisms [51]. One possibility to provide a purer test of conceptual adaptation would be to use a cross-modal adaptation paradigm [21,23]. Another possibility would be to manipulate the purported sex of each speaker while controlling for acoustics (e.g. labelling voices as male or female). Each of these possibilities could provide convergent evidence regarding the role of conceptual versus perceptual adaptation mechanisms. Second, future research could explicitly probe the cumulative nature of aftereffects to better understand these mechanisms.

More generally, our findings provide a foundation for future research to probe the efficacy of harnessing adaptation to mitigate biases. Existing evidence has used sensory adaptation to combat gender and sexual orientation-linked biases [38]. As such, sensory adaptation may be used to develop interventions to minimize known interpersonal biases such as voice-based discrimination in job hiring, leadership selection and criminal sentencing [10,11,72]. Although the study of evaluative aftereffects is still quite new, continued work in this area may unlock theoretical and practical knowledge about consequential judgements drawn from the human voice.

## Data Availability

All data, code and materials for Studies 1–4 are publicly available on OSF [75].

## Footnotes

There is some evidence that women’s faces are judged as more attractive when they deviate from the average [73,74], and that men’s and women’s voices are judged as more attractive when they are more typical [60].

Although these adaptors are more accurately described as hyper-typical (i.e. above the average *f*_o_ for women), we refer to them as gender-typical because they are shifted in a manner consistent with our beliefs about women and men (e.g. that women have high-pitched voices and men have low-pitched voices).

Future research could benefit from testing adaptation using a within-subjects, rather than between-subjects, design. This decision was made primarily to reduce participant fatigue which is common in studies with many trials. However, within-subject designs offer important controls for variation across participants.

## References

[1] Ambady N , Rosenthal R . 1992 Thin slices of expressive behavior as predictors of interpersonal consequences: a meta-analysis. Psychol. Bull. **111** , 256–274. (10.1037/0033-2909.111.2.256)

[2] Todorov A , Olivola CY , Dotsch R , Mende-Siedlecki P . 2015 Social attributions from faces: determinants, consequences, accuracy, and functional significance. Annu. Rev. Psychol. **66** , 519–545. (10.1146/annurev-psych-113011-143831) 25196277

[3] Lavan N . 2023 How do we describe other people from voices and faces? Cognition **230** , 105253. (10.1016/j.cognition.2022.105253) 36215763 PMC7618126

[4] Ostrega J , Feinberg DR . 2023 Topic analysis reveals first impressions of voices. PsyArXiv.

[5] Apicella CL , Feinberg DR . 2009 Voice pitch alters mate-choice-relevant perception in hunter-gatherers. Proc. Royal Soc. B. **276** , 1077–1082. (10.1098/rspb.2008.1542) PMC2679070 19129125

[6] Collins SA , Missing C . 2003 Vocal and visual attractiveness are related in women. Anim. Behav. **65** , 997–1004. (10.1006/anbe.2003.2123)

[7] McAleer P , Todorov A , Belin P . 2014 How do you say “hello”? Personality impressions from brief novel voices. PLoS ONE. **9** , e90779. (10.1371/journal.pone.0090779) 24622283 PMC3951273

[8] Miyake K , Zuckerman M . 1993 Beyond personality impressions: effects of physical and vocal attractiveness on false consensus, social comparison, affiliation, and assumed and perceived similarity. J. Pers. **61** , 411–437. (10.1111/j.1467-6494.1993.tb00287.x) 8246108

[9] Anderson RC , Klofstad CA . 2012 Preference for leaders with masculine voices holds in the case of feminine leadership roles. PLoS ONE. **7** , e51216. (10.1371/journal.pone.0051216) 23251457 PMC3520981

[10] Tigue CC , Borak DJ , O’Connor JJM , Schandl C , Feinberg DR . 2012 Voice pitch influences voting behavior. Evol. Hum. Behav. **33** , 210–216. (10.1016/j.evolhumbehav.2011.09.004)

[11] Chen D , Halberstam Y , Yu ACL . 2016 Perceived masculinity predicts U.S. Supreme Court outcomes. PLoS One **11** , e0164324. (10.1371/journal.pone.0164324) 27737008 PMC5063312

[12] Webster MA . 2012 Evolving concepts of sensory adaptation. F1000 Biol. Rep. **4** , 21. (10.3410/B4-21) 23189092 PMC3501690

[13] Anstis S , Verstraten FAJ , Mather G . 1998 The motion aftereffect. Trends Cogn. Sci. **2** , 111–117. (10.1016/s1364-6613(98)01142-5) 21227087

[14] Leopold DA , O’Toole AJ , Vetter T , Blanz V . 2001 Prototype-referenced shape encoding revealed by high-level aftereffects. Nat. Neurosci. **42** , 89–94. (10.1038/82947) 11135650

[15] Webster MA , Kaping D , Mizokami Y , Duhamel P . 2004 Adaptation to natural facial categories. Nature **428** , 557–561. (10.1038/nature02420) 15058304

[16] D’Alessandro LM , Norwich KH . 2009 Loudness adaptation measured by the simultaneous dichotic loudness balance technique differs between genders. Hear. Res. **247** , 122–127. (10.1016/j.heares.2008.10.009) 19027058

[17] Phillips DP , Scovil SJ , Carmichael ME , Hall SE . 2007 Adaptation of central pitch-specific mechanisms. Perception **36** , 918–930. (10.1068/p5581) 17718369

[18] Latinus M , Belin P . 2011 Human voice perception. Curr. Biol. **21** , R143–R145. (10.1016/j.cub.2010.12.033) 21334289

[19] Zäske R , Schweinberger SR , Kawahara H . 2010 Voice aftereffects of adaptation to speaker identity. Hear Res. **268** , 38–45. (10.1016/j.heares.2010.04.011) 20430084

[20] Bestelmeyer PEG , Rouger J , DeBruine LM , Belin P . 2010 Auditory adaptation in vocal affect perception. Cognition. **117** , 217–223. (10.1016/j.cognition.2010.08.008) 20804977

[21] Skuk VG , Schweinberger SR . 2013 Adaptation aftereffects in vocal emotion perception elicited by expressive faces and voices. PLoS ONE. **8** , e81691. (10.1371/journal.pone.0081691) 24236215 PMC3827484

[22] Hubbard DJ , Assmann PF . 2013 Perceptual adaptation to gender and expressive properties in speech: the role of fundamental frequency. J. Acoust. Soc. Am. **133** , 2367–2376. (10.1121/1.4792145) 23556602

[23] Little AC , Feinberg DR , Debruine LM , Jones BC . 2013 Adaptation to faces and voices: unimodal, cross-modal, and sex-specific effects. Psychol. Sci. **24** , 2297–2305. (10.1177/0956797613493293) 24068117

[24] Mullennix JW , Johnson KA , Topcu-Durgun M , Farnsworth LM . 1995 The perceptual representation of voice gender. J. Acoust. Soc. Am. **98** , 3080–3095. (10.1121/1.413832) 8550934

[25] Schweinberger SR , Casper C , Hauthal N , Kaufmann JM , Kawahara H , Kloth N , Robertson DMC , Simpson AP , Zäske R . 2008 Auditory adaptation in voice perception. Curr. Biol. **18** , 684–688. (10.1016/j.cub.2008.04.015) 18450448

[26] Zäske R , Schweinberger SR . 2011 You are only as old as you sound: auditory aftereffects in vocal age perception. Hear. Res. **282** , 283–288. (10.1016/j.heares.2011.06.008) 21771649

[27] Valentine T . 1991 A unified account of the effects of distinctiveness, inversion, and race in face recognition. Q. J. Exp. Psychol. A. **43** , 161–204. (10.1080/14640749108400966) 1866456

[28] Latinus M , McAleer P , Bestelmeyer PEG , Belin P . 2013 Norm-based coding of voice identity in human auditory cortex. Curr. Biol. **23** , 1075–1080. (10.1016/j.cub.2013.04.055) 23707425 PMC3690478

[29] Yovel G , Belin P . 2013 A unified coding strategy for processing faces and voices. Trends Cogn. Sci. **17** , 263–271. (10.1016/j.tics.2013.04.004) 23664703 PMC3791405

[30] Lavner Y , Rosenhouse J , Gath I . 2001 The prototype model in speaker identification by human listeners. Int. J. Speech Technol. , 63–74.

[31] Posner MI , Keele SW . 1968 On the genesis of abstract ideas. J. Exp. Psychol. **77** , 353–363. (10.1037/h0025953) 5665566

[32] Halberstadt J , Rhodes G . 2000 The attractiveness of nonface averages: implications for an evolutionary explanation of the attractiveness of average faces. Psychol. Sci. **11** , 285–289. (10.1111/1467-9280.00257) 11273386

[33] Sutherland CAM , Young AW , Mootz CA , Oldmeadow JA . 2015 Face gender and stereotypicality influence facial trait evaluation: counter-stereotypical female faces are negatively evaluated. Br. J. Psychol. **106** , 186–208. (10.1111/bjop.12085) 25168952

[34] Vingilis-Jaremko L , Maurer D . 2013 The influence of averageness on children’s judgments of facial attractiveness. J. Exp. Child Psychol. **115** , 624–639. (10.1016/j.jecp.2013.03.014) 23708730

[35] Apicella CL , Little AC , Marlowe FW . 2007 Facial averageness and attractiveness in an isolated population of hunter-gatherers. Perception **36** , 1813–1820. (10.1068/p5601) 18283931

[36] Repp BH . 1997 The aesthetic quality of a quantitatively average music performance: two preliminary experiments. Music Percept. **14** , 419–444. (10.2307/40285732)

[37] Bruckert L , Bestelmeyer P , Latinus M , Rouger J , Charest I , Rousselet GA , Kawahara H , Belin P . 2010 Vocal attractiveness increases by averaging. Curr. Biol. **20** , 116–120. (10.1016/j.cub.2009.11.034) 20129047

[38] Lick DJ , Johnson KL . 2014 Recalibrating gender perception: face aftereffects and the perceptual underpinnings of gender-related biases. J. Exp. Psychol. Gen. **143** , 1259–1276. (10.1037/a0034516) 24079449

[39] Lick DJ , Johnson KL . 2016 Straight until proven gay: a systematic bias toward straight categorizations in sexual orientation judgments. J. Pers. Soc. Psychol. **110** , 801–817. (10.1037/pspa0000052) 27281352

[40] Titze IR . 1994 Principles of voice production. Prentice Hall.

[41] Feinberg DR , DeBruine LM , Jones BC , Perrett DI . 2008 The role of femininity and averageness of voice pitch in aesthetic judgments of women’s voices. Perception **37** , 615–623. (10.1068/p5514) 18546667

[42] Skuk VG , Schweinberger SR . 2014 Influences of fundamental frequency, formant frequencies, aperiodicity, and spectrum level on the perception of voice gender. J. Speech Lang. Hear Res. **57** , 285–296. (10.1044/1092-4388(2013/12-0314) 23882002

[43] Kreiman J , Sidtis D . 2011 Foundations of voice studies: an interdisciplinary approach to voice production and perception. John Wiley & Sons. (10.1002/9781444395068) See https://onlinelibrary.wiley.com/doi/book/10.1002/9781444395068

[44] Leopold DA , Rhodes G , Müller KM , Jeffery L . 2005 The dynamics of visual adaptation to faces. Proc. R. Soc. B. **272** , 897–904. (10.1098/rspb.2004.3022) PMC1564098 16024343

[45] Judd CM , Westfall J , Kenny DA . 2017 Experiments with more than one random factor: designs, analytic models, and statistical power. Annu. Rev. Psychol. **68** , 601–625. (10.1146/annurev-psych-122414-033702) 27687116

[46] Aguinis H , Gottfredson RK , Culpepper SA . 2013 Best-practice recommendations for estimating cross-level interaction effects using multilevel modeling. J. Manage. **39** , 1490–1528. (10.1177/0149206313478188)

[47] Arend MG , Schäfer T . 2019 Statistical power in two-level models: a tutorial based on Monte Carlo simulation. Psychol. Methods. **24** , 1–19. (10.1037/met0000195) 30265048

[48] Lorah J . 2018 Effect size measures for multilevel models: definition, interpretation, and TIMSS example. Large-Scale Assess. Educ. **6** , 1–11. (10.1186/s40536-018-0061-2)

[49] Snijders T a. B . 2005 Power and sample size in Multilevel linear models. In Encyclopedia of Statistics in Behavioral Science (eds BS Everitt , DC Howell ), p. 15701573, vol. 3. Wiley. See 10.1002/0470013192.bsa492

[50] Westfall J , Kenny DA , Judd CM . 2014 Statistical power and optimal design in experiments in which samples of participants respond to samples of stimuli. J. Exp. Psychol. Gen. **143** , 2020–2045. (10.1037/xge0000014) 25111580

[51] Nussbaum C , von Eiff CI , Skuk VG , Schweinberger SR . 2022 Vocal emotion adaptation aftereffects within and across speaker genders: roles of timbre and fundamental frequency. Cognition **219** , 104967. (10.1016/j.cognition.2021.104967) 34875400

[52] Cohen J . 2013 Statistical power analysis for the behavioral sciences. Academic Press. (10.4324/9780203771587) See https://www.taylorfrancis.com/books/9781134742707

[53] Boersma P , Weenink D . 2023 Praat: doing phonetics by computer. See http://praat.org/

[54] R Core Team . 2020 R: A Language and Environment for Statistical Computing. Vienna, Austria: R Foundation for Statistical Computing. See https://www.R-project.org

[55] Bates D , Mächler M , Bolker B , Walker S . 2015 Fitting linear mixed-effects models using lme4. J. Stat. Softw. **67** , 1–48. (10.18637/jss.v067.i01)

[56] Kuznetsova A , Brockhoff PB , Christensen RHB . 2017 lmerTest package: tests in linear mixed effects models. J. Stat. Soft. **82** , 1–26. (10.18637/jss.v082.i13)

[57] Tingley D , Yamamoto T , Hirose K , Keele L , Imai K . 2014 Mediation: R package for causal mediation analysis. J. Stat. Softw. **59** , 1–38. (10.18637/jss.v059.i05)26917999

[58] Puts DA . 2005 Mating context and menstrual phase affect women’s preferences for male voice pitch. Evol. Hum. Behav. **26** , 388–397. (10.1016/j.evolhumbehav.2005.03.001)

[59] Puts DA , Barndt JL , Welling LLM , Dawood K , Burriss RP . 2011 Intrasexual competition among women: vocal femininity affects perceptions of attractiveness and flirtatiousness. Pers. Indiv. Differ. **50** , 111–115. (10.1016/j.paid.2010.09.011)

[60] Bauer DJ , Preacher KJ , Gil KM . 2006 Conceptualizing and testing random indirect effects and moderated mediation in multilevel models: new procedures and recommendations. Psychol. Methods. **11** , 142–163. (10.1037/1082-989X.11.2.142) 16784335

[61] Rhodes G , Jeffery L , Watson TL , Clifford CWG , Nakayama K . 2003 Fitting the mind to the world: face adaptation and attractiveness aftereffects. Psychol. Sci. **14** , 558–566. (10.1046/j.0956-7976.2003.psci_1465.x) 14629686

[62] Gregory Jr SW , Gallagher TJ . 2002 Spectral analysis of candidates’ nonverbal vocal communication: predicting U.S. presidential election outcomes. Soc. Psychol. Q. **65** , 298–308. (10.2307/3090125)

[63] Klofstad CA . 2016 Candidate voice pitch influences election outcomes. Polit. Psychol. **37** , 725–738. (10.1111/pops.12280)

[64] Klofstad CA , Anderson RC , Peters S . 2012 Sounds like a winner: voice pitch influences perception of leadership capacity in both men and women. Proc. Royal Soc. B. **279** , 2698–2704. (10.1098/rspb.2012.0311) PMC3350713 22418254

[65] Mayew WJ , Parsons CA , Venkatachalam M . 2013 Voice pitch and the labor market success of male chief executive officers. Evol. Hum. Behav. **34** , 243–248. (10.1016/j.evolhumbehav.2013.03.001)

[66] Klofstad CA , Anderson RC . 2018 Voice pitch predicts electability, but does not signal leadership ability. Evol. Hum. Behav. **39** , 349–354. (10.1016/j.evolhumbehav.2018.02.007)

[67] Re DE , O’Connor JJM , Bennett PJ , Feinberg DR . 2012 Preferences for very low and very high voice pitch in humans. PLoS ONE. **7** , e32719. (10.1371/journal.pone.0032719) 22403701 PMC3293852

[68] O’Connor JJM , Feinberg DR . 2012 The influence of facial masculinity and voice pitch on jealousy and perceptions of intrasexual rivalry. Pers. Indiv. Differ. **52** , 369–373. (10.1016/j.paid.2011.10.036)

[69] O’Connor JJM , Pisanski K , Tigue CC , Fraccaro PJ , Feinberg DR . 2014 Perceptions of infidelity risk predict women’s preferences for low male voice pitch in short-term over long-term relationship contexts. Pers. Indiv. Differ. **56** , 73–77. (10.1016/j.paid.2013.08.029)

[70] Zuckerman M , Driver RE . 1988 What sounds beautiful is good: the vocal attractiveness stereotype. J. Nonverbal Behav. **13** , 67–82. (10.1007/BF00990791)

[71] Ostrega J , Little AC , Feinberg DR . 2020 Adaptation effects of voice-pitch on attractiveness judgements. PsyArXiv.

[72] Schroeder J , Epley N . 2015 The sound of intellect: speech reveals a thoughtful mind, increasing a job candidate’s appeal. Psychol. Sci. **26** , 877–891. (10.1177/0956797615572906) 25926479

[73] Perrett DI , May KA , Yoshikawa S . 1994 Facial shape and judgements of female attractiveness. Nature **368** , 239–242. (10.1038/368239a0) 8145822

[74] Perrett DI , Burt DM , Penton-Voak IS , Lee KJ , Rowland DA , Edwards R . 1999 Symmetry and human facial attractiveness. Evol. Hum. Behav **20** , 295–307. (10.1016/S1090-5138(99)00014-8)

[75] OSF . Social Evaluative Implications of Sensory Adaptation to Human Voices. See https://osf.io/msnke/?view_only=34a23c90fc634cb581eeb4d1c14af975 10.1098/rsos.231348 PMC10966390 38544561

